# The Co-Selection of Fluoroquinolone Resistance Genes in the Gut Flora of Vietnamese Children

**DOI:** 10.1371/journal.pone.0042919

**Published:** 2012-08-24

**Authors:** Le Thi Minh Vien, Ngo Ngoc Quang Minh, Tang Chi Thuong, Huynh Duy Khuong, Tran Vu Thieu Nga, Corinne Thompson, James I. Campbell, Menno de Jong, Jeremy J. Farrar, Constance Schultsz, H. Rogier van Doorn, Stephen Baker

**Affiliations:** 1 Oxford University Clinical Research Unit, Wellcome Trust Major Overseas Programme, Ho Chi Minh City, Vietnam; 2 Centre for Tropical Diseases, University of Oxford, Oxford, United Kingdom; 3 Children's Hospital, Ho Chi Minh City, Vietnam; 4 Department of Medical Microbiology, Academic Medical Centre, University of Amsterdam, Amsterdam, The Netherlands; 5 Department of Global Health, Academic Medical Centre, University of Amsterdam, Amsterdam, The Netherlands; Louisiana State University and A & M College, United States of America

## Abstract

Antimicrobial consumption is one of the major contributing factors facilitating the development and maintenance of bacteria exhibiting antimicrobial resistance. Plasmid-mediated quinolone resistance (PMQR) genes, such as the *qnr* family, can be horizontally transferred and contribute to reduced susceptibility to fluoroquinolones. We performed an observational study, investigating the copy number of PMQR after antimicrobial therapy. We enrolled 300 children resident in Ho Chi Minh City receiving antimicrobial therapy for acute respiratory tract infections (ARIs). Rectal swabs were taken on enrollment and seven days subsequently, counts for *Enterobacteriaceae* were performed and *qnrA*, *qnrB* and *qnrS* were quantified by using real-time PCR on metagenomic stool DNA. On enrollment, we found no association between age, gender or location of the participants and the prevalence of *qnrA*, *qnrB* or *qnrS*. Yet, all three loci demonstrated a proportional increase in the number of samples testing positive between day 0 and day 7. Furthermore, *qnrB* demonstrated a significant increase in copy number between paired samples (*p*<0.001; Wilcoxon rank-sum), associated with non-fluoroquinolone combination antimicrobial therapy. To our knowledge, this is the first study describing an association between the use of non-fluoroquinolone antimicrobials and the increasing relative prevalence and quantity of *qnr* genes. Our work outlines a potential mechanism for the selection and maintenance of PMQR genes and predicts a strong effect of co-selection of these resistance determinants through the use of unrelated and potentially unnecessary antimicrobial regimes.

## Introduction

Appropriate antimicrobial usage in the management of infectious diseases is an important issue for patients and administering clinicians. The issue is particularly relevant in many low and middle income countries, where the sale of antimicrobials is often unrestricted [1], [2], [3]. It is apparent that the sustained usage of some antimicrobial classes has, over time, reduced the effect of these drugs on once susceptible pathogens and encouraged the selection of resistant bacteria [4], [5], [6]. The implications of antimicrobial resistant pathogenic bacteria are clear, including treatment failure, increasing treatment costs and protracted therapy [7]. Furthermore, antimicrobial resistant organisms, or the mobile elements carrying the resistance genes, can be transmitted to other individuals and organisms, respectively, increasing the proportion of antimicrobial resistant organisms circulating in the general population [8], [9].

Plasmid-mediated quinolone resistance (PMQR) determinants, in the form of *qnr* genes, were first reported in 1998 in a clinical *K. pneumoniae* isolate from a patient with a urinary tract infection in the USA [10]. In less than ten years, the reported prevalence of *qnr* genes (*qnrA*, *qnrB*, *qnrS*, *qnrC* and *qnrD*) has increased dramatically , reported globally across a spectrum of bacterial species, such as *E. coli*, *Klebsiella pneumoniae* [11], [12], [13]. The phenotype conferred by Qnr determinants is generally low level resistance to fluoroquinolones, yet they appear to encourage additional fluoroquinolone resistance mechanisms, subsequently resulting in high level resistance [10]. Our work in Ho Chi Minh City (HCMC), Vietnam, and the work of others, has shown that *qnr* genes are common in commensal organisms and are carried by mobile elements harbouring multiple antimicrobial resistance genes [14], [15], [16]. Furthermore, we have suggested that *qnr* genes are co-selected by the use of non-quinolone antimicrobials and we have hypothesized that the use of antimicrobials, other than fluoroquinolones, plays a critical role in the selection and maintenance of *qnr* genes in the commensal human gut flora.

Acute respiratory tract infections (ARIs) are the most common infection in children in developed and developing countries and are the leading cause of death in children less than five years of age [17]. A meta-analysis highlighted the global ARI disease burden, , estimating that 1.9 million (95% Confidence Interval [CI] 1.6–2.2 million) children died from ARIs in 2000, of which 70% of deaths were in Africa and Southeast Asia [17]. Viruses are the dominant etiologic agents of ARIs, responsible for approximately 60% of all cases [17]. However, because an etiological diagnosis is not normally established quickly, antimicrobials are typically the mainstay of empirical ARI treatment [18]. Here, we have developed several real-time PCR assays to detect and enumerate *qnr* genes and quantified these loci in paired rectal swabs from children with symptoms of ARI prior to, and after seven days of, antimicrobial treatment.

## Materials and Methods

### Participants, enrolment and ethical approval

Children presenting to the outpatient department of Children's Hospital 1 in HCMC between April and November 2009 were eligible for enrolment in this study. The inclusion criteria were: less than 16 years of age, symptoms of ARI, not admitted to hospital, living in HCMC, prepared to come back for follow-up at day 7 and informed written consent given by a parent or a legal guardian. The age, demographics and prescribed antimicrobials were recorded at the time of enrolment (Day 0), after the consultation and a written prescription from the clinician making the medical assessment. All antimicrobials were prescribed at the discretion of the treating clinicians. Rectal swabs from all recruited children were collected on enrolment (prior to antimicrobial therapy) and on Day 7 (after antimicrobial therapy). Rectal swabs were stored in 1 ml of sterile saline, which is commonly used for transporting samples and making dilutions of *Enterobacteriaceae*. These solutions were processed for microbiology on the day of collection and were stored at −80°C until molecular analysis. This study was performed in accordance with the declaration of Helsinki and was granted ethical approval by Children's Hospital 1 in HCMC, Vietnam and the Oxford Tropical Research Ethics Committee (OxTREC), in the United Kingdom. Informed written consent was given by a parent or a legal guardian.

### Sample processing and colony counting

Ten-fold serial dilutions (from 10^−1^ to 10^−8^) were made from all rectal swabs on the day of collection. Fifty microlitres of each dilution was inoculated onto MacConkey agar (Oxoid, UK) plates and incubated at 37°C overnight. The number of bacterial colonies on each plate was enumerated and plates containing between 30 to 300 colonies were used to calculate the CFU ml^−1^.

After microbiological assessment, the fecal samples were processed in batches (Day 0 and Day 7 of the same children) to ensure limited experimental variation. We compared the quality of extracted DNA using three DNA extraction methods, DNAeasy (QIAGEN, USA), QIAam DNA Stool mini kit (QIAGEN, USA) and the nucliSENS EasyMag system (bioMérieux, Mercy l'Étoile, France). The nucliSENS EasyMag system demonstrated the highest quantity, quality and reproducibility. As a result, after addition of the internal control, total nucleic acid was extracted from 200 µl of stool using the nucliSENS EasyMag system, according to the manufacturer's instructions, and was eluted in 50 µl of elution buffer.

### Primer design and quantitative real-time PCR

The nucleotide sequences of all available full length alleles for *qnrA*, *qnrB* and *qnrS* were retrieved from NCBI [19], and aligned using Vector NTI (Invitrogen). Primers and probes for each of the three loci were designed using Primer Express version 2.0 (Applied Biosystems Inc.). The specificity of primers and probes was assessed using BLASTn against the NCBI database and were synthetized by Sigma-Proligo (Singapore). The primers and probes were as follows: *qnrA*; qnrA_RT_F; CAGTTTCGAGGATTGCAGTT, qnrA_RT_R, CCTGAACTCTATGCCAAAGC, qnrA_probe; Fam - AAGGGTGYCACTTCAGCTATGCC – Tamra; *qnrB*; qnrB_RT_F; CAGATTTYCGCGGCGCAAG; qnrB_RT_R; TTCCCACAGCTCRCAYTTTTC, qnr_probe; Fam - CGCACCTGGTTTTGYAGYGCMTATATCAC – Tamra; *qnrS*; qnrS_RT_F; TCAAGTGAGTAATCGTATGTA, qnrS_RT_R; GTCTGACTCTTTCAGTGAT, qnrS_probe; Fam – CCAGCGATTTTCAAACAACTCAC – Tamra. An internal control consisting of PhHV DNA was added to each sample. Primers and probe for Phocid Herpes Virus (PhHV), which were used as an internal control, were as previously described [20].

Real-time PCR was performed using HotStart Taq polymerase (QIAGEN) on a Chromo4 Real-time PCR machine (Bio-Rad, Hercules, CA, USA). Reactions were performed in 25 µl volumes, containing 2.5 µl of 10× buffer, 5 mM of MgCl_2_, 500 nM of dNTPs, 400 nM of each primer, 100 nM of probe, 1 U of HotStart Taq polymerase and 5 µl of DNA template. Optimized real-time PCR conditions were: one cycle of 95°C for 15 minutes, followed by 45 cycles of 30 seconds at 95°C, 30 seconds at annealing temperature for primer pair and probe and 30 seconds at 72°C. The optimized annealing temperatures were: 52°C for *qnrA*, 54°C for *qnrB* and 55°C for *qnrS*. A positive PCR signal was defined as any Ct (threshold cycle) value <40.

### Standard curves

The target genes (*qnrA1*, *qnrB1* and *qnrS1*) were amplified in their entirety with previously described primers [21], [22], [23] and cloned into pCR2.1-TOPO and transferred into *E. coli* TOP10 cells using the TOPO TA Cloning kit (Invitrogen, UK), according to the manufacturer's instructions. Plasmids containing a cloned insertion were detected by blue-white colony selection on Luria-Bertani (LB) agar supplemented with 50 mg/L ampicillin and 40 mg/L X-gal. Plasmid DNA was extracted using the QIAprep Miniprep (QIAGEN), digested with *XhoI* restriction endonuclease (*qnrA1*, *qnrB1* and *qnrS1* do not have *XhoI* restriction sites), purified (QIAquick PCR Purification Kit, QIAGEN) and quantified using a NanoDrop ND-1000 Spectrophotometer (NanoDrop Technologies, Inc). Concentrations were then converted to copy number. Ten-fold serial dilutions of plasmid DNA containing the cloned target insert were used as an external standard for all quantitative real-time RT PCR experiments.

### PCR Reproducibility, linearity and efficiency

The reproducibility of each assay was assessed by calculating the co-efficient of variance (CV%). The CV calculates the deviation in the Ct values of selected plasmid DNA concentrations across multiple amplifications, both in the same run and in different runs. Intra-assay reproducibility was assessed by comparing the Ct values generated in the same run of four replicates of each plasmid concentration. Inter-assay reproducibility was assessed by comparing the Ct values generated by four replicates of each plasmid concentration over a period of four days. Linearity was assessed by the Ct values of 10-fold serial dilutions of plasmid DNA containing cloned target sequences (concentrations 5×10^0^–5×10^8^) and was calculated by linear regression. Efficiency was again calculated from the slope of the standard curve, using the formula: Efficiency = 10^(−1/slope)^−1, according to the methods of Rasmussen *et al* [24].

### Internal extraction and positive amplification controls

For optimizing each of the *qnr* real-time PCR assays, isolates that we have previously identified as being *qnr*-positive were used as positive controls, these were, *qnrA1*; *Klebsiella pneumoniae* K222Ca, *qnrB1*; *Klebsiella pneumoniae* K281An and *qnrS1*; *Klebsiella pneumoniae* K35N [25]. Efficiency of nucleic acid extraction and real-time PCR amplification was monitored in all reactions by inclusion of the PhHV internal control [20]. Aliquots of post culture supernatant containing PhHV were prepared. The precise amount of virus added to the fecal samples was assessed by PCR titration during the standard curves experiments. The final dilution and volume was selected on the basis that it reproducibly produced a Ct value within the range of 30–33, equating to approximately copies of PhHV added to each sample prior to nucleic acid extraction. Any amplification run that did not produce a Ct value between 30 and 33 for PhHV was discarded. Positive amplification controls with known Ct values for each gene and ‘no-template negative control’ were included in each amplification run.

### Statistical analysis

The gene copy numbers and the CFU ml^−1^ of *Enterobacteriaceae* in each sample was calculated and logarithmically transformed for analysis using MS excel (Microsoft). The demographic data, prevalence of *qnr* genes and copy number of *qnr* genes between Day 0 and Day 7 were compared using the Fisher's exact test, McNemar's test and Wilcoxon's rank-sum test, respectively. Results were interpreted as statistically significant when *p*<0.05. All analyses were performed using R version 2.12.0 program (Foundation for Statistical Computing, Vienna, Austria).

## Results

### Validation of quantitative real-time PCR assays for *qnr* genes

We developed three real-time PCR assays to quantify the copy number of *qnrA*, *qnrB* and *qnrS*. Plasmids containing cloned *qnrA*, *qnrB* and *qnrS* target sequences were used as positive controls for quantifying amplification. PCR amplicons for cloning were generated using genomic DNA extracted from bacterial isolates previously found to be positive for one of the three target loci [25]. The real-time PCR assays were tested on nucleic acid extracted from 45 enteric organisms previously found to be *qnr* negative including, *E. coli*, *Shigella spp.*, *Salmonella spp.*, *Citrobacter spp*., *Enterobacter spp*., *Pantoea spp.*, *Proteus mirabilis* and *Klebsiella spp*. None of the three loci demonstrated any unspecific amplification with any target nucleic acid from any of the tested organisms.

To assess the potential for cross hybridization between the three gene targets, each PCR assay was performed using template DNA prepared from each of the *qnrA*, *qnrB* and *qnrS* positive control strains. These experiments were repeated in triplicate. Each replicate for all PCR assays produced an identical result and each assay was specific for the intended target. The detection limit of each of the three assays was calculated on the basis of fifty individual Ct values for the diluted plasmid DNA samples, produced from ten replicates over five days that consistently generated a positive signal in ≥95% of reactions. These concentrations were <50 copies per reaction of the cloned target sequences of all three targets.

### Reproducibility and linearity

Table 1 shows the results from a series of consecutive standard curve experiments. These data demonstrate the overall performance, intra-assay variation and inter-assay variation of the three real-time PCR assays. The intra-assay and inter-assay co-efficient of variance across the three targets ranged from 0.56–2.72% (>97% reproducibility) and 1.27–2.96% (>97% reproducibility), respectively, with target copy numbers ranging from 5×10^1^ to 5×10^7^ copies per reaction. Linearity was assessed by a standard curve produced from the Ct values generated from amplification of the 5×10^7^ to the 5×10^1^ copies per reaction (i.e. seven data points) and was calculated by linear regression. The linear regressions of the three standard curves were R^2^ = 0.999 for *qnrA*, R^2^ = 0.999 for *qnrB* and R^2 = ^0.998 for *qnrS*, indicating a strong linear correlation between Ct value and target gene concentration. The efficiencies of the amplifications were 87% for *qnrA* (95CI; 85–90%), 91% for *qnrB* (95CI; 89–92%) and 87% *qnrS* (95CI; 81–92%).

**Table 1:** The validation of quantitative real-time PCR assays targeting *qnrA*, *qnrB* and *qnrS*.

Variable	Target copy number						
	5×10^7^	5×10^6^	5×10^5^	5×10^4^	5×10^3^	5×10^2^	50
***qnrA***							
Ct value	12.32	16.05	20.58	23.44	27.53	31.3	34.58
Intra-assay CV%	0.75	0.91	0.79	0.74	1.46	2.00	2.72
Ct value	12.41	16.37	20.40	23.42	27.35	31.3	34.39
Inter-assay CV%	1.43	1.57	1.45	2.55	2.90	2.86	2.96
***qnrB***							
Ct value	12.41	16.40	20.60	23.17	27.44	31.35	34.52
Intra-assay CV%	0.65	0.76	0.56	1.22	1.28	1.47	1.91
Ct value	12.41	16.35	20.47	23.46	27.20	31.45	34.37
Inter-assay CV%	1.83	2.19	2.46	1.86	2.12	2.45	2.83
***qnrS***							
Ct value	13.09	16.86	20.12	23.69	27.77	30.72	34.12
Intra-assay CV%	1.38	0.65	1.43	2.02	1.62	1.13	1.59
Ct value	13.28	16.99	20.77	24.18	27.30	31.40	34.56
Inter-assay CV%	2.67	1.27	2.18	2.12	2.09	1.70	2.45

The inter-assay and intra-assay variability were checked on bacterial nucleic acid extracted from pure culture with and without internal control PhHV. No differences were observed between these two batches.

CV: Coefficient of variance.

### Baseline study population characteristics

Three hundred children were enrolled for the purposes of this study, resulting in a total of 600 rectal swabs. Bacterial colony counting and quantitative real-time PCR for all three loci were performed on all 600 samples. For subsequent data analysis we required accurate PCR amplification and a bacterial colony count on all 600 stool samples and antimicrobial usage data from each of the 300 enrolees. We rejected 37 paired samples for the purposes of our analysis. From these rejected paired samples, 19 samples had a negative internal control, 16 samples had less than 30 colonies cultured and two enrolees did not have available antimicrobial usage data. Therefore, paired data from 263 enrolled children were available for analysis.

The resulting baseline data for the detection of *qnrA*, *qnrB* and *qnrS* on Day 0 from 263 children is shown in Table 2. The children were resident in 18 different districts in HCMC, which we divided into three groups based on population density, an *a priori* variable that we believed may confound the relationship between local *qnr* gene exposure and circulation (Table 2). The median age of enrolees was 12 months (range: 2 months to 12 years) and the male to female ratio was 1∶0.83. On Day 0, we detected *qnrA* in 12 samples (4.5%), *qnrB* in 116 samples (44.1%) and *qnrS* in 196 samples (74.5%). We found no significant association between age, gender or population density of the participants with *qnr* gene prevalence on Day 0 using Fisher's exact test (Table 2).

**Table 2:** Baseline demographic characteristics of participants and the prevalence of *qnr* genes prior to antimicrobial therapy.

Variable	*qnrA* positive	*qnrB* positive	*qnrS* positive
	(n = 263)	(n = 263)	(n = 263)
**Sex**			
Male (n = 144)	6 (4.2%)	67 (46.5%)	111 (77.1%)
Female (n = 119)	6 (5%)	49 (41.2%)	85 (71.4%)
**Age**			
<1 (n = 66)	3 (4.5%)	37 (56.1%)	57 (86.4%)
1 (n = 75)	5 (6.7%)	34 (45.3%)	55 (73.3%)
2 (n = 51)	1 (2%)	20 (39.2%)	34 (66.7%)
3 (n = 43)	2 (4.7%)	19 (44.2%)	31 (72.1%)
>3 (n = 28)	1 (3.6%)	6 (21.4%)	19 (68%)
**Population density** *			
Low (n = 95)	5 (5.3%)	44 (46.3%)	72 (75.8%)
Medium (n = 116)	3 (2.6%)	48 (41.4%)	86 (74%)
High (n = 52)	4 (7.7%)	24 (46.2%)	38 (73.1%)

* Districts within HCMC with population densities <10,000 (low), 10,000–30000 (medium) and >30,000 people/km^2^ (high).

### Prevalence and quantification of *qnr* genes in stool samples from children with ARIs

We compared the proportion of rectal swabs demonstrating a PCR amplification signal for *qnrA*, *qnrB* and *qnrS* using paired samples taken on Day 0 and Day 7 (Table 3). All three loci demonstrated a proportional increase in the number of samples testing positive for the *qnr* loci. The percentage of samples testing positive increased by 4.2%, 24% and 4.2% for *qnrA*, *qnrB* and *qnrS*, respectively. The comparative difference between these samples was assessed by McNemar's test; only *qnrB* demonstrated a statistically significant increase (*p*<0.001).

**Table 3:** Comparison of *qnr* gene prevalence, *qnr* copy number and CFU ml^−1^ of *Enterobacteriaceae* in stool samples on enrolment and seven days after enrolment.

Variable	Day 0	Day 7	*p* value
**Prevalence of ** ***qnr*** ** genes (%)**			
*qnrA*	12/263 (4.5%)	23/263 (8.7%)	0.054
*qnrB*	116/263 (44.1%)	179/263 (68.1%)	<0.001*
*qnrS*	196/263 (74.5%)	207/263 (78.7%)	0.272
***Enterobacteriaceae*** ** CFU ml^−1^, median (range)**			
	1.3×10^7^	3.3×10^7^	<0.001*
	(800–9×10^8^)	(780–5×10^11^)	
***qnr*** ** gene copy number ml^−1^, median (range)**			
*qnrA*	7.4×10^4^	2.2×10^4^	0.102
	(2.6×10^3^–5.2×10^7^)	(2.7×10^3^–2.8×10^7^)	
*qnrB*	4.8×10^4^	1.3×10^5^	<0.001*
	(2.6×10^3^–7.8×10^7^)	(2.7×10^3^–4.2×10^10^)	
*qnrS*	2.8×10^5^	1.1×10^6^	<0.001*
	(2.7×10^3^–7.9×10^8^)	(4×10^3^–2.2×10^9^)	
***qnr*** ** gene copy number per ** ***Enterobacteriaceae*** ** CFU ml^−1^, median (range)**			
*qnrA*	1.8×10^−3^	9.1×10^−4^	0.102
	(2×10^−5^–1.6)	(3.6×10^−6^–0.289)	
*qnrB*	1.2×10^−3^	4.5×10^−3^	<0.001*
	(3×10^−6^–485)	(7×10^−8^–2.1×10^4^)	
*qnrS*	1.7×10^−2^	2.9×10^−2^	0.226
	(1×10^−5^–1065)	(5×10^−7^–2.8×10^3^)	

Evaluation of the prevalence of *qnr* genes, gene copy number, CFU ml^−1^ and *qnr* gene copy number per CFU. Statistical significance was assessed by McNemar's test and paired Wilcoxon rank-sum test.

* Statistically significant (*p*<0.05).

To compare the number of *Enterobacteriaceae* in the intestinal flora before and after the use of antimicrobials, the CFU ml^−1^ of *Enterobacteriaceae* on MacConkey agar were calculated. We did not formally assess a coefficient of variance for the colony counts from rectal swabs. However, whilst developing this technique for the purposes of this investigation, 20 samples were tested in triplicate and the counts were found to have tolerable variation (+/−10% of the primary value). The median CFU ml^−1^ in paired samples demonstrated a significant increase from 1.26×10^7^ (range; 8×10^2^ to 9×10^8^) on Day 0 to 3.26×10^7^ (range 7.8×10^2^ to 5×10^11^) on Day 7 (*p*<0.001, paired Wilcoxon rank-sum) (Table 3).

**Figure 1:**
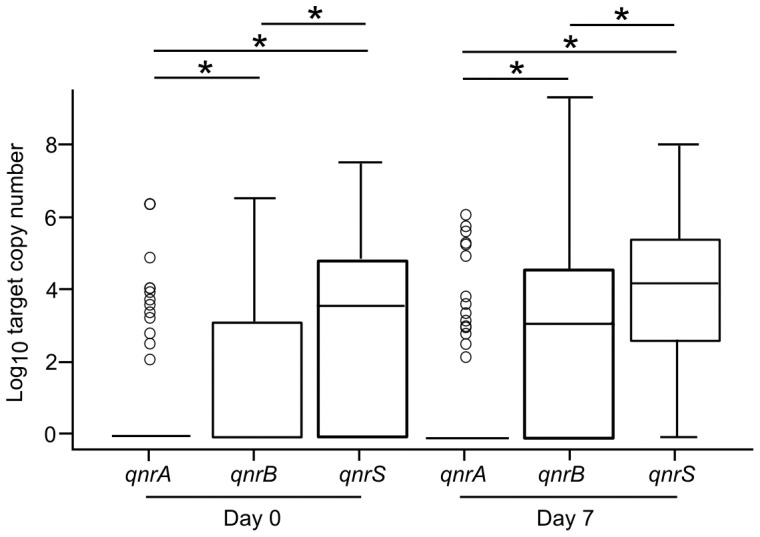
The median gene copy number of *qnrA*, *qnrB and qnrS* on enrolment and after antimicrobial therapy. Box plots showing the median and interquartile ranges of *qnrA*, *qnrB and qnrS* gene copy numbers in stool samples collected from children with ARIs on enrolment (Day 0) and after antimicrobial treatment (Day 7). Statistical significance between the *qnr* genes was calculated using the paired Wilcoxon rank-sum test; significant variation in gene copy number between the *qnr* genes is denoted at the head of the figure (*), all *p* values<0.001.

By the use of a standard curve on each independent run, we calculated the copy number of *qnrA*, *qnrB* and *qnrS* in the 526 stool samples. The median copy numbers of *qnrA*, *qnrB* and *qnrS* on Day 0 were 7.4×10^4^ (range: 2.6×10^3^ to 5.2×10^7^), 4.8×10^4^ (range: 2.6×10^3^ to 7.8×10^7^) and 2.8×10^5^ (range: 2.7×10^3^ to 7.9×10^8^), respectively. The median copy numbers of *qnrA*, *qnrB* and *qnrS* on Day 7 were 2.2×10^4^ (range: 2.7×10^3^ to 2.8×10^7^), 1.3×10^5^ (range: 2.7×10^3^ to 4.2×10^10^) and 1.1×10^6^ (range: 4×10^3^ to 2.2×10^9^), respectively. The *qnrS* gene had the highest copy numbers of the three-*qnr* loci on both day 0 and day 7 (Figure 1). After adjusting copy number for *Enterobacteriaceae* CFU ml^−1^, *qnrB* demonstrated a significant increase between Day 0 and Day 7 (*p*<0.001, paired Wilcoxon rank-sum) (Table 3).

### Comparing antimicrobial usage and *qnr* gene copy number

We stratified the copy numbers of *qnrA*, *qnrB* and *qnrS* in the 263 enrolled children by their prescribed antimicrobial treatment regimen (Table 4). Twelve different antimicrobials, belonging to three different classes; β-lactam and β-lactamase inhibitor combination (amoxicillin-clavulanic acid), cephalosporins (cefaclor, cefadroxil, cefixime, ceftriaxone, cefuroxime, cefpodoxime) and macrolides (azithromycin, erythromycin, roxithromycin, spiramycin) were used to treat enrolled participants. Notably, no children were prescribed fluoroquinolones. Seven different antimicrobial regimens were prescribed, in the form of mono-therapy or in a combination of two or more antimicrobials (Table 4).

**Table 4:** Comparison of gene copy number for *qnrA*, *qnrB* and *qnrS* before and after three different alternative treatment regimes.

	Day 0	Day 7	*p* value^a^	*p* value^b^
	Median copy number (range)	Median copy number (range)		
**Group 1: Co-amoxiclav (n = 114)**				
*qnrA*	0 (0–4.3×10^3^)	0 (0–4.3×10^5^)	0.01*	0.016*
*qnrB*	0 (0–1.3×10^6^)	967 (0–5.6×10^8^)	<0.001*	<0.001*
*qnrS*	1.2×10^3^ (0–1.6×10^7^)	1.5×10^4^ (0–4.7×10^7^)	<0.001*	0.391
CFU ml^−1^	4×10^5^ (80–1.9×10^7^)	1.8×10^6^ (67–1.9×10^9^)	<0.001*	n/a
**Group 2: Cephalosporin (n = 86)**				
*qnrA*	0 (0–1×10^6^)	0 (0–439)	0.402	0.529
*qnrB*	62 (0–1.6×10^6^)	126 (0–5.4×10^6^)	0.097	0.196
*qnrS*	915 (0–3×10^6^)	2.7×10^3^ (0–5.4×10^6^)	0.365	0.512
CFU ml^−1^	7.6×10^5^ (40–1.9×10^7^)	1.5×10^6^ (300–2.5×10^10^)	0.001*	n/a
**Group 3: Co-amoxiclav + Cephalosporin (n = 36)**				
*qnrA*	0 (0–3.8×10^4^)	0 (0–5.6×10^5^)	1	0.933
*qnrB*	30 (0–4.6×10^5^)	4.4×10^3^ (0–2.2×10^6^)	<0.001*	<0.001*
*qnrS*	2.9×10^3^ (0–7.5×10^6^)	7.6×10^4^ (0–9×10^6^)	0.017*	0.187
CFU ml^−1^	1.3×10^6^ (4.2×10^3^–1.6×10^7^)	3.5×10^6^ (39–7.2×10^7^)	0.024*	n/a

* *p*<0.05. Statistical significance calculated by paired Wilcoxon rank-sum test.

a *p* value calculated by comparing *qnr* gene copy number between Day 0 and Day 7.

b *p* value calculated by comparing *qnr* gene copy number between Day 0 and Day 7 after adjusting for *Enterobacteriaceae* CFU ml^−1^.

We analysed the CFU ml^−1^ of the *Enterobacteriaceae* with each treatment regimen between the two time points. Three groups (co-amoxiclav, cephalosporin only and both combined) demonstrated a significant increase in *Enterobacteriaceae* CFU ml^−1^ between the two time points (*p*<0.05) (Table 4). Again we adjusted the gene copy numbers from day 0 and day 7 for *Enterobacteriaceae* CFU ml^−1^ within the treatment regimes. There was a significant increase in copy number between Day 0 and Day 7 for the *qnrA* and *qnrB* loci in those treated with co-amoxiclav and co-amoxiclav combined with a cephalosporin (Table 4).

## Discussion

PMQR genes stimulate low level resistance to fluoroquinolones, yet their ability to facilitate other fluoroquinolone resistance mechanisms and their promiscuity threatens the long-term efficacy of the fluoroquinolones [10]. There has been intense investigation of PMQR genes across a wide diversity of bacterial species, with some studies focusing on detecting *qnr*, *aac(6′)-Ib-cr* and *qepA* genes using real-time PCR or by pyrosequencing in clinical isolates [26], [27], [28], [29]. Here, for the first time, we have used real-time PCR to detect and quantify *qnr* genes in nucleic acid extracted directly from rectal swabs of patients treated with antimicrobials.

This observational real-time PCR investigation for *qnrA*, *qnrB* and *qnrS* on paired stool samples from children with ARIs living in HCMC reveals several new insights into the prevalence and selection of *qnr* genes in this location. Notably, the prevalence of *qnrB* increased between the day of enrolment and seven days after antimicrobial therapy, even after stratification by the number of *Enterobacteriaceae*. Our data suggest that this increase is related to selection of *qnrB* through antimicrobial exposure, either by direct selection or enrichment of existing *qnrB* containing organisms in the gastrointestinal tract. The uptake and maintenance of *qnrB* is likely through contaminated food or constant exposure to organisms with this gene from known environmental sources [30], [31], [32]. Moreover, co-existence of *qnrB* with other resistance genes, such as *bla_CTX-M-14_* or *bla_CTX-M-15_*, on the same plasmid is a well-known phenomenon [33], [34]. In our previous study of *qnr* genes in organisms isolated in the hospital and the community in HCMC, we found that 11%, 12% and 45% of bacterial isolates from hospitalised individuals were positive for *qnrA*, *qnrB* and *qnrS*, respectively, and 0.7%, 0.5%, 12% from community isolates were positive for *qnrA*, *qnrB* and *qnrS*, respectively [25]. Here, we detected a substantially higher prevalence of the PMQR genes than before. Explanations for this disparity include the use of updated primers (i.e. the ability to detect newer alleles); differences in the target populations (bacterial isolates vs. metagenomic approach on stool samples); an increased sensitivity of real-time vs. conventional PCR method; and a difference of four years, which may reflect a natural increase in the circulation of organisms (and people) carrying PMQR genes.

There have been several reports related to antimicrobial usage and an increase in resistant bacteria, yet this is the first describing evidence of the co-selection of PMQR genes in this manner [35], [36]. Both *qnrB* and *qnrS* demonstrated significant copy number increases between Day 0 and Day 7. Yet, none of the individuals enrolled in this study were treated with fluoroquinolones and instead were prescribed amoxicillin/clavulanic acid, macrolides or cephalosporins. This increase in copy number over the two time points, apparently via exposure to these antimicrobials, particularly amoxicillin/clavulanic acid or amoxicillin/clavulanic acid combined with a cephalosporin, substantiates our co-selection hypothesis. Indeed, additional selection is the most likely mechanism, as PMQR plasmids frequently contain other resistance genes, particularly determinants encoding resistance to β-lactams [37], [38], [39], [40], [41]. Our currently unpublished data related to PMQR plasmids and the genetic background of *qnrS1* containing mobile elements in HCMC reveals a predominant *qnrS1*-containing transposon type carrying the *bla_LAP-2_* gene, which also encodes resistance to β-lactams. Additional reports have also shown an intimate association between *qnrA* and *qnrB* with ESBL genes [14], [15], [42], [43], [44].

It is predicted that the number and structure of the bacteria population in the human gut depends on health status, diet and antimicrobial usage [45], [46], [47]. Here, by using MacConkey agar, we investigated potential differences in the number of *Enterobacteriaceae* between two time points [48]. It is known that the diversity and the abundance of organisms in stool is great and includes a variety of *Bacteroides*, *Clostridia*, *Fusobacteria* and *Peptostreptococci* [49], [50], all of which may contribute to the overall differences observed in PMQR gene prevalence and copy number. However, we observed a significant increase in the CFU ml^−1^ of *Enterobacteriaceae* between the two time points, implying a strong selection of these organisms, potentially through antimicrobial resistance enrichment. We found a significant increase in CFU ml^−1^ in rectal swabs between Day 0 and Day 7 related to three antimicrobial treatment regimens: amoxicillin/clavulanic acid only, a cephalosporin only, or amoxicillin/clavulanic acid combined with a cephalosporin. However, only amoxicillin/clavulanic acid seemed to influence *qnr* gene copy number (before and after adjusting for CFU ml^−1^ of *Enterobacteriaceae*), implying that the increase of *qnr* genes is not only a consequence of *Enterobacteriaceae* enrichment but these genes may also be shared by other bacterial species within the intestinal tract [33]. Our results clearly show a major shift in *qnr* gene copy numbers after antimicrobial therapy, yet our findings are limited by a lack of control group who did not receive antimicrobials. The results presented here warrant asking additional questions related to the effect of antimicrobials on the presence and maintenance of *qnr* genes and other antimicrobial resistance genes in the gut flora. We are currently longitudinally following a cohort of children in HCMC over a two-year period with and without antimicrobial treatment to address natural fluctuations of resistance genes and changes in *Enterobacteriaceae*.

In conclusion, our data demonstrate an increasing prevalence of *qnrB* and an increasing quantity of the *qnrB* and *qnrS* genes in the stools of children with ARIs between enrolment and after seven days treatment with non-fluoroquinolone antimicrobials. This is the first study describing an association between the use of non-quinolone antimicrobials and the increasing relative prevalence in *qnr* gene copy number in the gut flora. Our work highlights the rampant nature of PMQR genes in this locality and suggests aggressive co-selection of these resistance determinants through the use of unrelated and potentially unnecessary antimicrobial treatment regimes.
